# Absorption of Polyunsaturated Fatty Acid (PUFA) Is Related to IgG Blood Levels of Neonatal Pigs during the First 48 Hours Postpartum

**DOI:** 10.1155/2020/3813250

**Published:** 2020-02-06

**Authors:** Kateryna Pierzynowska, Jarosław Woliński, Björn Weström, Radosław Jazwiec, Halyna Shmigel, Stefan G. Pierzynowski

**Affiliations:** ^1^Department of Animal Physiology, Kielanowski Institute of Animal Physiology and Nutrition, Jablonna 05110, Poland; ^2^Department of Biology, Lund University, Lund 22362, Sweden; ^3^Anara AB & SGPlus, Trelleborg 23132, Sweden; ^4^Institute of Biochemistry and Biophysics, Polish Academy of Sciences, Warsaw 02106, Poland; ^5^Vitananano Sp. z o.o. & PROF, Lublin 20492, Poland; ^6^Department of Biochemistry and Biotechnology, Vasyl Stefanyk Precarpathian National University, Ivano-Frankivsk 76000, Ukraine; ^7^Department of Medical Biology, Institute of Rural Health, Lublin 20090, Poland

## Abstract

The current study is aimed at highlighting the impact of enterally or parenterally applied immunoglobulins (Igs) on polyunsaturated fatty acid (PUFA) absorption in newborn pigs. Piglets were chosen as the appropriate model since they are born agammaglobulinemic and any effects of Ig addition can thus be easily monitored. Twenty-one, new born piglets were used in the study. Plasma levels of PUFAs, ARA, DHA, and EPA dropped (similarly to that seen in human infants) by between 40 and 50% in newborn, unsuckled piglets fed an infant formula for 48 h. However, piglets fed the same infant formula but supplied with immunoglobulins (Igs) either orally, by feeding piglets with swine or bovine colostrum, or intravenously, by i.u.a. (intraumbilical artery) infusion of swine or human Ig preparations or swine serum, demonstrated improved growth and PUFA levels similar to those observed at birth. The significant positive correlation was found between the body weight gain, as well as levels of ARA and EPA, and plasma immunoglobulins concentration. These results indicate the importance of the presence of Ig in the blood for appropriate absorption of dietary PUFAs and probably other nutrients in newborn piglets. This may have an impact on the dietary guidelines for human neonates, especially those born prematurely with low plasma Ig levels, since PUFAs are important factors for brain development in early life.

## 1. Introduction

The omega-3 long-chain polyunsaturated fatty acid (PUFA), docosahexaenoic acid (DHA), is an important component for the development of the nervous system and the maintenance of cognitive function [1, 2]. This is particularly true in preterm infants, since the majority of foetal nervous system structural development, such as cortical growth and gyrification, occurs throughout the third trimester [3]. Along with essential fatty acids, linoleic acid (LA) and alpha linoleic acid (ALA), as well as omega-6 fatty acids such as arachidonic acid (ARA), PUFAs also contribute to a number of physiological functions, including that of immunomodulation [4, 5]. However, PUFAs are poorly digested and thus poorly absorbed in preterm infants due to the immaturity of the gastrointestinal system and (developmental) exocrine pancreatic insufficiency [6–9].

To minimize deficiencies in PUFAs, infant formulas are often supplemented with LA, and important PUFAs such as DHA and ARA [10, 11]. However, plasma concentrations of DHA and ARA in preterm infants drop precipitously soon after birth and remain low, despite massive parenteral and enteral nutritional support [4, 5]. Since the fat in infant formulas cannot be provided in an “absorbable” form due to the risk of inflammation caused by fatty acids, the fat must be provided in a stable triglyceride form. The triglycerides in turn require hydrolysis by pancreatic lipase to fatty acids and monoglycerides before absorption and utilization in the body [12].

Purified plasma Igs for intravenous use are broadly used in clinics to treat symptomatically different sickness, to modulate various immunological pathways at both the humoral and cellular levels, including inflammation, antigen presentation, cell growth, and apoptosis [13]. Administration of exogenous Igs demonstrated a high efficacy in the treatment of sepsis and acute lung injury in adults [14, 15]. Since preterm neonates are at increased risk of life-threatening infections and problems with regard to development of the nervous system [16–19], the potential advantage of treatment with exogenous Igs could be even greater than in adults. Unfortunately, the effects of Igs on the development of the central nervous system are poorly understood.

In nature, transfer of Igs from the colostrum to the blood in newborn piglets takes place in the gut mucosa, during the first 24 hours of life [20]. Previous studies from our lab have shown that immunoglobulin supplementation in newborn, healthy piglets who are naturally agammaglobulinemic at birth could ensure appropriate brain development in terms of cognitive function and improvements in neuronal plasticity [21]. Since ARA and DHA are recognised as important, indispensable factors for brain development, we have decided to explore whether Igs can affect the absorption of these PUFAs.

The main aim of the study was to highlight the dependency of PUFA absorption on the presence of immunoglobulins in the blood of newborn piglets. Piglets were chosen as an appropriate model since they belong to the ungulate family and are born agammaglobulinemic, and thus the effects of Ig addition can be easily monitored.

## 2. Materials and Methods

All experimental procedures were approved by the University of Lund Ethics Review Committee on Animal Experiments (approval No. M142-14). All efforts were made to minimize animals' suffering.

### 2.1. Animals

The experiment was carried out on crossbred ((Yorkshire x Swedish Landrace) x Hampshire) pigs (Sus scrofa domesticus) obtained from a SPF local herd (Vindfälle 810, 268 68 Röstånga, Sweden). Twenty-one piglets, a mixture of males and females, were randomly chosen to partake in the experiment, directly after parturition from six sows. The piglets were weighed, marked, and housed under a heating lamp (150 W). The piglets were then transported to the animal facility of the Dept. of Biology, Lund University.

### 2.2. Experimental Design

Sample size was estimated using G^∗^Power software, version 3.1.9.4 [22] for a one-way ANOVA at *α* = 0.05 with 95% power, assuming *f* (effect size) = 2 and *SD* = 20, for seven study groups.

All piglets were implanted with a silastic catheter in one of their umbilical arteries, under anesthesia with 0.5-1.5% air mixture of Fluothane (Zeneca, Gothenburg, Sweden) and O_2_ as a carrier gas, at approximately 0.5-1 l/min in a close-circuit respiratory system (Komesaroff Medical Developments, Melbourne, Australia).

**Figure 1:**
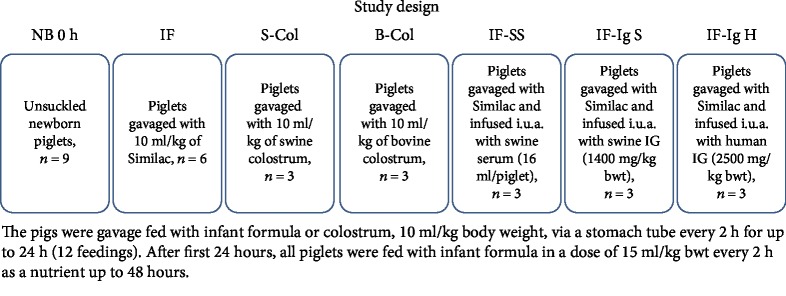
Study design. Newborn nonsuckled piglets (NB) (*n* = 9); piglets fed for 48 hours with infant formula (IF) (*n* = 6); piglets fed for 48 hours with either swine colostrum (S-Col) (*n* = 3) or bovine colostrum (B-Col) (*n* = 3); piglets fed infant formula supplemented i.u.a. with either swine serum (IF+SS) (*n* = 3), swine immunoglobulins (IF+IgS) (*n* = 3), or human immunoglobulins (IF+IgH) (*n* = 3).

After baseline blood sampling, the piglets were randomized into seven experimental groups. Group IF (*n* = 6) was fed an infant formula containing ARA (0.014 g/100 ml) and DHA (0.009 g/100 ml) (Similac 24 Special Care with iron manufactured by Abbott Nutrition, Lake Forest, IL, USA). Group S-Col (*n* = 3) was fed swine colostrum (EPA, DHA, and ARA content of 0.98, 0.90, and 0.44 g/100 g FA, respectively [23]). Group B-Col (*n* = 3) was fed bovine colostrum (EPA, DHA, and ARA content of 0.28, 0.02, and 0.48 g/100 g FA, respectively [24]). Group IF-SS (*n* = 3) was fed the infant formula and given an intraumbilical artery (i.u.a.) infusion of swine serum. The swine serum was injected in three doses, at 5, 13, and 21 h after birth, 4, 5, and 7 ml/kg bwt, respectively. Group IF-Ig S (*n* = 3) was fed the infant formula and given an i.u.a. infusion of purified porcine immunoglobulin (piglets were infused, via the umbilical vein, 1540 mg/kg bwt–25 ml of sterile Ig preparation/kg bwt). Group IF-Ig H (*n* = 3) was fed the infant formula and given an i.u.a. infusion of purified human immunoglobulin (piglets were infused, via the umbilical artery, 2500 mg/kg bwt–25 ml of sterile Ig preparation/kg bwt). All piglets were fed their respective diets via a stomach tube in a volume of 10 ml/kg body weight, every two hours for up to 12 hours (six feedings). After this, the piglets were fed exclusively with the infant formula in a volume of 15 ml/kg bwt every two hours for up to 48 hours (to the end of experiment). The piglets in particular groups were kept together under a heating lamp. In addition, unsuckled newborn piglets (Group NB, *n* = 9) that were not subjected to any form of treatment were sacrificed within 1 h after birth and included as controls. The information on detailed dosing and source of immunoglobulins, as well as on dietary PUFA levels, is presented in Table 1. The schematic study design is provided in Figure 1.

### 2.3. Blood Sample Collection

Baseline (0 hours) and final (48 hours) blood samples were collected via the umbilical artery catheter and transferred to BD Vacutainer® glass tubes coated with lithium heparin (BD Diagnostics, New Jersey, USA). The collected samples were immediately placed on ice before they were centrifuged at 3000 x g for 15 minutes at 4°C, and plasma was separated and stored at -80°C until further analysis.

### 2.4. Quantification of Free DHA, ARA, and EPA in Plasma

For the measurement of free PUFA (*μ*g/ml), 100 *μ*l of the blood sample from each piglet was precipitated using 100 *μ*l of internal standard (deuterated ARA, DHA, and EPA obtained from Sigma-Aldrich) solution in IPA (POCh). After that, it was acidified by addition of 100 *μ*l of 2% HCOOH. Samples were subsequently extracted using 600 *μ*l of hexane (Avantor Baker) and the organic layer was evaporated under nitrogen. Samples were solubilized in 100 *μ*l of 5% NH4OH in 65% MeOH and 10 *μ*l was injected into LC/MS. Samples were then analysed using Waters Xevo TQ-S triple quadrupole MS coupled with Waters Acquity UPLC chromatograph. The column used was an ACQUITY UPLC BEH C18 Column, 1.7 *μ*m, 2.1 mm × 50 mm thermostated in 60°C. Mobile phase A was 0.1% NH4OH in MQ water; mobile phase B was pure LC/MS grade ACN (Avantor Baker). A linear gradient was used, starting from 40% to 95% B over 1.7 min. MS was operating in negative MRM mode. Parameters were as follows: capillary voltage 3.2, desolvation temperature 500°C, desolvation gas (nitrogen) flow 900 **l**/h, and cone gas flow 150 **l**/h. Calibration and control samples were prepared by spiking PUFA standards into 45 mg/ml fatty acid-free BSA (Sigma-Aldrich, St. Louis, MO, USA) solution in 0.9% NaCl.

### 2.5. Autopsy and Sample Collection

Following the 48 hour feeding period, the piglets were euthanized by a single dose of sodium pentobarbiturate (100 mg/kg), i.u.a. At autopsy specimens from the stomach, the duodenum, ileum, and pancreas were collected, flushed with ice-cold saline, and immediately fixed in Bouin's solution for 48 hours. Samples were then dehydrated and embedded into paraffin, according to standard histological techniques. Prior to routine H&E (hematoxylin and eosin, Histolab, Gothenburg, Sweden) staining, tissue blocks were sliced into 5 *μ*m thick slices, using a rotor microtome. After staining, samples were dehydrated and mounted onto a slide, under a coverslip with DPX medium. A histological analysis was done on each sample using a light microscope (Olympus PROVIS, Tokyo, Japan).

### 2.6. Analyses of IgG Content

The concentration of swine, bovine, and human IgG (*μ*g/ml) in the plasma samples, as well as in the swine serum, bovine, and swine colostrum and the porcine Igs preparation, was analysed by a single radial immunodiffusion [25], using antiporcine IgG produced in rabbits (Sigma P0916) and purified porcine IgG as the standard (Sigma 14381), anti-bovine IgG produced in rabbits (Sigma B5645) and purified bovine IgG as the standard (Sigma I5506), anti-human IgG produced in goat (Sigma I1886) and purified human IgG as the standard (Sigma I4506, all Sigma-Aldrich, St. Louis, MO, USA), respectively.

### 2.7. Calculations and Statistics

Data are expressed as mean ± SD. An ANOVA, followed by a Tukey post hoc test for normally distributed datasets, and a Kruskal-Wallis test followed by Dunn's multiple comparison test for datasets with nonparametric distribution (GraphPad Prism, v 8.1.0) were used to assess statistical differences between groups. To assess data distribution, Shapiro-Wilk normality test was performed. Spearman's correlation was used to examine correlations between variables. Differences were considered significant if *p* ≤ 0.05.

## 3. Results

**Figure 2:**
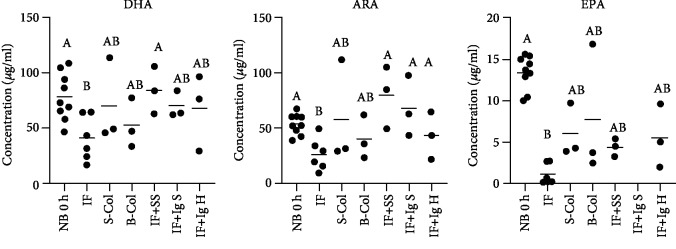
Blood levels of PUFA in piglets. Newborn nonsuckled piglets (NB) (*n* = 9); piglets fed for 48 hours with infant formula (IF) (*n* = 6); piglets fed for 48 hours with either swine colostrum (S-Col) (*n* = 3) or bovine colostrum (B-Col) (*n* = 3); piglets fed infant formula supplemented i.u.a. with either swine serum (IF+SS) (*n* = 3), swine immunoglobulins (IF+IgS), (*n* = 3), or human immunoglobulins (IF+IgH) (*n* = 3). Individual values are shown for each experimental group (*n* = 3‐9). Capital letters given with results mean significant differences within the dot columns when *p* < 0.05.

**Figure 3:**
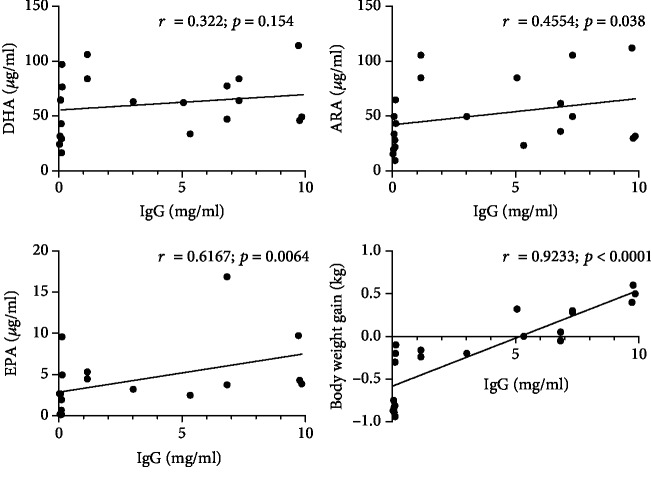
Scatterplots showing correlation between changes in piglets' body weight gain and plasma PUFA concentrations with IgG plasma concentrations; Spearman correlations (*r*). Data expressed as individual values at the end of the treatment period, for all study groups, except NB (newborn nonsuckled piglets).

Blood levels of DHA, ARA, and EPA in newborn unsuckled piglets (NB group) were around 78.5, 53.5, and 13.41 *μ*g/ml, respectively (Figure 2). In piglets fed for 48 hours exclusively with infant formula (IF group), a significant decline in blood DHA, ARA, and EPA was observed (*p* < 0.01) when compared to the NB group. The blood levels of DHA, ARA, and EPA in piglets which got any kind of immunoglobulin supplementation (S-Col, B-Col, IF+SS, IF+IgS, and IF+IgH groups) were not significantly different from those observed in the NB group (Figure 2). In piglets supplemented i.u.a. with swine serum (IF+SS group), the blood levels of both DHA and ARA, but not EPA, were significantly (*p* < 0.05) higher than those observed in the IF group (Figure 2). Piglets supplemented i.u.a. with swine immunoglobulins (IF+IgS group) also demonstrated significantly (*p* < 0.05) higher levels of ARA, but not DHA and EPA, when compared to the IF group (Figure 2). It is worth mentioning that significant positive correlation was found between the blood levels of ARA and EPA and plasma immunoglobulin concentration (Figure 3). No correlation was found between blood DHA level and plasma immunoglobulin concentration (Figure 3).

With regard to body weight gain, piglets in the IF group demonstrated a significant loss of body weight during the 48 hour feeding period when compared to the NB group (*p* < 0.05) (Table 2). In fact, only piglets from the S-Col and IF+IgS groups gained weight within the first 48 hours of the study, while those in the other experimental groups displayed either an absence of body weight gain or weight loss (Table 2). The significant positive correlation was found between the body weight gain and plasma immunoglobulin concentration (Figure 3).

The histological examination of the small intestine and pancreatic tissue did not reveal any significant differences between groups—data not shown.

## 4. Discussion

Several recent studies have shown that PUFA absorption and thus their availability are critical factors for brain development [26] in newborns. Their benefits for the growth and maturation of an infant's brain and visual acuity are well known, and the mechanisms of such effects have been widely investigated. PUFA supplementation has also been shown to improve both cognitive [27] and psychomotor development [28] in preterm human infants.

Recently, pig models have shown that the initial high levels of PUFA observed at birth significantly decrease directly after birth and their recovery towards initial baseline values is very difficult to achieve by simple supplementation of the infant formula with DHA or ARA [29]. At the same time, the maintenance of optimal lipid composition and metabolism in the brain is not only important during the early stages of brain development and maturation from gestation through childhood [30, 31], but also is crucial for healthy brain aging [32–34].

So the question remains, why is supplementation of infant formula with DHA and ARA only slightly effective in returning PUFA levels to those observed at birth [4, 5, 35] and how can one improve the absorption of PUFAs in the gastrointestinal tract of newborns?

Since a previous study from our lab [21] demonstrated that parenteral or enteral supplementation with IgG improves brain development and behavior in a newborn pig model, born agammaglobulinemic, we decided to investigate how the absorption of PUFAs is related to the presence of IgG in the blood and gastrointestinal tract.

A very narrow time frame exists (around 36 hours) during which the possible effects of immunoglobulins on the absorption of PUFAs can be investigated, since it has to be done when the piglets still have a so-called “open gut.” Gut closure is the phenomenon which occurs approximately 36 hours after birth, prior to this, the gut enterocytes [36, 37] are capable of absorbing intact IgG from the sow's colostrum. In fact, gut absorption is the only way in which ungulates are able to get IgG into their circulation.

The current experiment indicated that the presence of Igs in the blood is important for ensuring that PUFAs are absorbed from the gut. Independently, immunoglobulins offered directly by feeding with colostrum or injected i.u.a., either as a preparation of swine or human IgG, as well swine serum, ensure higher levels of DHA, ARA, and EPA in the blood, 48 hours after birth. While in the piglets, not obtaining any form of immunoglobulins, ARA, DHA, and EPA levels remained between 40 and 50% lower than that observed in newborn piglets or piglets treated with immunoglobulins.

It is interesting that the levels of EPA, which is synthesized in the neonate, are lower in hypoimmunogenic animals than in animals obtaining i.u.a. treatment with IgG. We know that EPA is synthetized from the absorbed alpha-linolenic acid (ALA), and this process' efficiency is very low. However, the IgG treatment led to the increase in EPA plasma levels only after 48 hours and probably could regulate the infant's EPA synthesis. It seems that plasma immunoglobulins not only ensure the absorption of PUFAs from gut but also affect essential fatty acid interactions and/or synthesis as manifested by the increased level of EPA.

It is also worth mentioning that PUFA has a wide range of metabolites, including eicosanoids, which are a large class of highly potent regulatory lipid hormones, possessing a wide range of biological functions. DHA and EPA are also parent molecules of specialized proresolving lipid mediators (SPMs). SPMs have anti-inflammatory and proresolving characteristics and include protectins, D-series resolvins, and maresins derived from DHA and EPA via cyclooxygenase and lipoxygenase pathways [38, 39]. Deficiencies in the levels of the n-3 PUFAs, DHA, and EPA, which are parent molecules of SPMs, could impair B-cell development, differentiation, and maturation, with further impairments in immunoglobulin synthesis [40, 41]. Thus, the development of adaptive immunity which takes place in infants could be impaired with an n-3 PUFA deficit. Considering these observations, the role of immunoglobulins becomes even more important.

The species specificity of immunoglobulins is less important since both human and bovine immunoglobulins affect PUFA absorption similarly to swine immunoglobulins. However, with regard to body weight gain, which is one of the main factors for the assessment of newborn pig development, as well as for human neonates, only high doses of swine immunoglobulins (swine colostrum feeding or i.u.a. injection of swine Igs preparation) appeared to be sufficient to ensure body weight gain in the preterm piglets. This finding could be explained by the inability of foreign immunoglobulins to influence the gut cellular structure [42] and brain-gut interactions [21, 43], in the same manner as swine Igs. It is worth pointing out that malabsorption of dietary PUFAs and evident growth retardation observed in preterm human infants [44] can be associated with impaired absorption of other nutrients, including macromolecules, as well as in Ig-deprived piglets. Thus, our present data supports the suggestions postulated by Ahmadi et al. [45] and Juhl et al. [46] about dietary supplementation of preterm children with bovine colostrum.

The data presented here might have implications for the formulation/revision of nutritional guidelines for preterm and newborn children, with special emphasis on the importance of immunoglobulins for appropriate PUFA (probably other nutrients as well) absorption and growth.

## 5. Conclusions

The study presented in the manuscript was planned and designed as an explorative experiment, carried out on the reliable agammoglobulinemic newborn pig model, in order to reveal whether the maternal immunoglobulins (specifically IgG) of different origin administered via different administration routes have any effect on the absorption of essential fatty acids. The main strength of this study is the novel approach to immunonoglobulins and their relationship to dietary fat absorption. Analysis of both n-3 and n-6 PUFAs in the agammaglobulinemic newborn pig model is another key strength of this study.

The main limitation of this study is the relatively small sample size and observational character of the findings. Further, long-term studies involving more animals and large panel of molecular techniques, which could help to elucidate mechanisms underlying the observed effects, are currently being planned. We hope that they will enable the verification and explanation of obtained results.

In summary, we identified a significant influence of immunoglobulin supplementation on PUFA absorption during early postnatal development.

## Figures and Tables

**Table 1:** IgG concentrations in swine and bovine colostrum, swine serum, and swine and human Ig preparations. Newborn nonsuckled piglets (NB) (*n* = 9); piglets fed for 48 hours with infant formula (IF), (*n* = 6). Piglets fed for 48 hours with either only swine colostrum (S-Col) (*n* = 3) or only bovine colostrum (B-Col) (*n* = 3). Piglets fed infant formula supplemented i.u.a. with either swine serum (IF-SS) (*n* = 3), swine immunoglobulins (IF-Ig S) (*n* = 3), or human immunoglobulins (IF-Ig H) (*n* = 3).

Group	Dose (mg/kg bwt)		
	Swine IG	Bovine IG	Human IG
NB 0 h	NA	NA	NA
IF	NA	NA	NA
S-Col	6052.80	NA	NA
B-Col	NA	6549.60	NA
IF+SS	403.52	NA	NA
IF+IgS	1540.08	NA	NA
IF +IgH	NA	NA	2500

NA: not applicable.

**Table 2:** Blood levels of IgG and PUFA and body weight gain in piglets. Newborn nonsuckled piglets (NB) (*n* = 9); piglets fed for 48 hours with infant formula (IF) (*n* = 6). Piglets fed for 48 hours alone either swine colostrum (S-Col) (*n* = 3) or bovine colostrum (B-Col) (*n* = 3). Piglets fed infant formula supplemented i.u.a. either with swine serum (IF+SS) (*n* = 3), swine immunoglobulins (IF+IgS) (*n* = 3), or human immunoglobulins (IF+IgH) (*n* = 3). Data are presented as mean ± SD, (*n* = 3‐9). a = bovine IgG, b = human IgG. Superscripted capital letters given with results imply significant differences within the columns when *p* < 0.05.

Groups	IgG (mg/ml)	Body weight gain (kg)
NB 0 h	0.087 ± 0.045	NA
IF	0.076 ± 0.024	−0.85 ± 0.07^A^
S-Col	9.77 ± 0.54	0.50 ± 0.08^B^
B-Col	6.33 ± 0.86(a)	0.00 ± 0.05^C^
IF+SS	1.78 ± 1.07	−0.20 ± 0.04^D^
IF+Ig S	6.56 ± 1.30	0.30 ± 0.02^E^
IF+Ig H	0.13 ± 0.02(b)	−0.20 ± 0.07^D^

NA: not applicable.

## Data Availability

The datasets generated and analysed during the current study are available from the corresponding authors on reasonable request.
